# How to Get an NIA Grant

**DOI:** 10.1093/geroni/igab046.1217

**Published:** 2021-12-17

**Authors:** Kenneth Santora

**Affiliations:** National Institute on Aging, Bethesda, Maryland, United States

## Abstract

Dr. Santora will provide an overview of the NIA application process and will share information on relevant policy changes.

